# Triethyl­ammonium (*S*)-(−)-*O*-[1-(2-naphth­yl)eth­yl] (4-meth­oxy­phen­yl)dithio­phospho­nate

**DOI:** 10.1107/S1600536811015820

**Published:** 2011-04-29

**Authors:** Samet Solak, Mehmet Karakuş, Barış Tercan, Tuncer Hökelek

**Affiliations:** a Department of Chemistry, Pamukkale University, 20017 Kınıklı, Denizli, Turkey; b Department of Physics, Karabük University, 78050 Karabük, Turkey; c Department of Physics, Hacettepe University, 06800 Beytepe, Ankara, Turkey

## Abstract

The crystal structure of the title compound, C_6_H_16_N^+^·C_19_H_18_O_2_PS_2_^−^, consists of the dithio­phospho­nate anions and the triethyl­ammonium cations, which are linked by N—H⋯S hydrogen bonds and weak C—H⋯O hydrogen bonds. In the anion, the benzene ring is oriented with respect to the naphthalene ring system at a dihedral angle of 24.92 (5)°. In the crystal, weak C—H⋯π inter­actions also occur.

## Related literature

For dithio­phospho­rus compounds and their complexes, see: Heiduc *et al.* (2006 ▶); Karakuş *et al.* (2007 ▶); Gataulina *et al.* (2008 ▶). For the roles of dithio­phospho­rus compounds in agricultural, industrial and medicinal products such as additives to lubricant oils, solvent extraction reagents for metals, floatation agents for minerals, pesticides and insecticides, see: Thomas *et al.* (2001 ▶); Gray *et al.* (2003 ▶). For the synthetic routes reported for dithio­phospho­rus-type ligands, see: Alberti *et al.* (2007 ▶). For the preparation of ferrocenyl and aryl­dithio­phospho­nates and their complexes with a range of transition metals, see: Gray *et al.* (2004 ▶). For bond-length data, see: Allen *et al.* (1987 ▶).
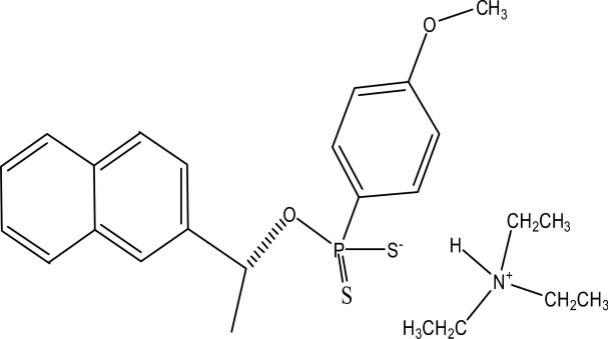


## Experimental

### 

#### Crystal data

C_6_H_16_N^+^·C_19_H_18_O_2_PS_2_^−^
*M* *_r_* = 475.62
Orthorhombic, 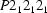

*a* = 9.3782 (3) Å
*b* = 12.3467 (5) Å
*c* = 21.9651 (8) Å
*V* = 2543.33 (16) Å^3^
*Z* = 4
Mo *K*α radiation
μ = 0.29 mm^−1^
*T* = 294 K
0.52 × 0.36 × 0.32 mm

#### Data collection

Bruker Kappa APEXII CCD area-detector diffractometer
Absorption correction: multi-scan (*SADABS*; Bruker, 2005 ▶) *T*_min_ = 0.862, *T*_max_ = 0.912
43596 measured reflections
6343 independent reflections
5946 reflections with *I* > 2σ(*I*)
*R*_int_ = 0.030

#### Refinement

*R*[*F* ^2^ > 2σ(*F* ^2^)] = 0.036
*wR*(*F* ^2^) = 0.094
*S* = 1.06
6343 reflections
289 parameters
H atoms treated by a mixture of independent and constrained refinement
Δρ_max_ = 0.78 e Å^−3^
Δρ_min_ = −0.26 e Å^−3^
Absolute structure: Flack (1983 ▶), 2752 Friedel pairs
Flack parameter: −0.01 (6)

### 

Data collection: *APEX2* (Bruker, 2007 ▶); cell refinement: *SAINT* (Bruker, 2007 ▶); data reduction: *SAINT*; program(s) used to solve structure: *SHELXS97* (Sheldrick, 2008 ▶); program(s) used to refine structure: *SHELXL97* (Sheldrick, 2008 ▶); molecular graphics: *ORTEP-3 for Windows* (Farrugia, 1997 ▶); software used to prepare material for publication: *WinGX* (Farrugia, 1999 ▶) and *PLATON* (Spek, 2009 ▶).

## Supplementary Material

Crystal structure: contains datablocks I, global. DOI: 10.1107/S1600536811015820/xu5201sup1.cif

Structure factors: contains datablocks I. DOI: 10.1107/S1600536811015820/xu5201Isup2.hkl

Supplementary material file. DOI: 10.1107/S1600536811015820/xu5201Isup3.cml

Additional supplementary materials:  crystallographic information; 3D view; checkCIF report

## Figures and Tables

**Table 1:** Hydrogen-bond geometry (Å, °) *Cg*1 and *Cg*2 are the centroids of the C1–C6 and C10–C13/C18/C19 rings, respectively.

*D*—H⋯*A*	*D*—H	H⋯*A*	*D*⋯*A*	*D*—H⋯*A*
N1—H1⋯S2^i^	0.84 (3)	2.52 (3)	3.2911 (17)	154 (2)
C20—H20*A*⋯O1	0.97	2.56	3.505 (2)	166
C7—H7*B*⋯*Cg*2^ii^	0.96	2.90	3.658 (3)	137
C24—H24*B*⋯*Cg*1^iii^	0.97	2.79	3.750 (2)	171

Symmetry codes: (i) 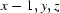; (ii) 
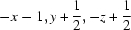
; (iii) 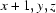.

**Fig. 1.**
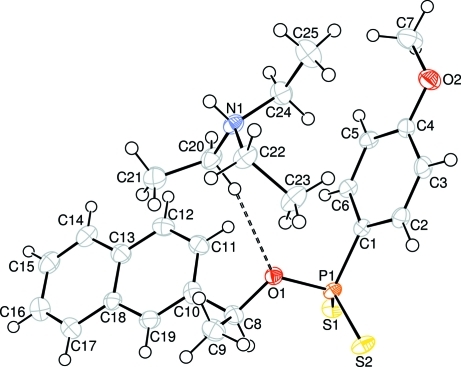
The molecular structure of the title molecule with the atom-numbering scheme. Displacement ellipsoids are drawn at the 50% probability level. C—H···O hydrogen bond is shown as dashed line.

**Fig. 2.**
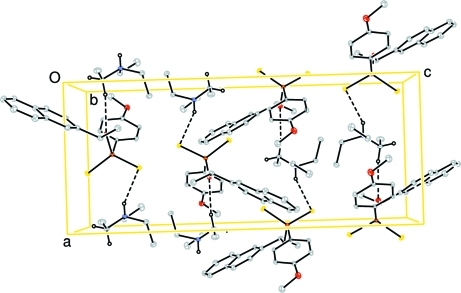
A view of the crystal packing of the title compound. The C-H···O and N-H···S hydrogen bonds are shown as dashed lines [H-atoms not involved in hydrogen bonding have been omitted for clarity].

## supplementary crystallographic information

### Comment

Dithiophosphorus compounds and their complexes have been widely investigated in last decades (Heiduc *et al.*, 2006; Karakuş *et al.*, 2007; Gataulina *et al.*, 2008). They have been utilized in agricultural, industrial and medicinal products such as additive to lubricant oils, solvent extraction reagents for metals, floatation agents for minerals, pectidites and insecticides (Thomas *et al.*, 2001; Gray *et al.*, 2003). For example, tin diphenyldithiophosphinato complexes show an antiproliferation activity towards certain leukaemia cells (Gray *et al.*, 2003). In general, dithiophosphorus type ligands are not commercially available, but a few synthetic routes were reported in the literature (Alberti *et al.*, 2007). When compared to the other dithiophosphorus derivatives, there is very limited research on dithiophosphonates in the last century, due to the difficulties in sythesizing these compounds. Recently, ferrocenyl and aryldithiophosphonates and their complexes with a range of transition metals were prepared by Woolins *et al.* (Gray *et al.*, 2003; Gray *et al.*, 2004). The present study was undertaken to ascertain the crystal structure of the title compound to contribute to this relatively less developed area.

The title compound consists of a dithiophosphonate bridged napthylethyl and methoxyphenyl groups and a triethylammonium moiety linked by a C-H···O hydrogen bond (Table 1 and Fig. 1), where the bond lengths are close to standard values (Allen *et al.*, 1987).

An examination of the deviations from the least-squares planes through individual rings shows that rings A (C1—C6), B (C10—C13/C18/C19) and C (C13—C18) are planar. The naphthalene group, containing the rings B and C are also nearly planar [with a maximum deviation of -0.022 (2) Å for atom C13] with a dihedral angle of B/C = 1.67 (7)°. Ring A is oriented with respect to the planar naphthalene group at a dihedral angle of 24.92 (5)°.

In the crystal, C—H···O and N-H···S hydrogen bonds link the molecules into chains along [100] (Table 1 and Fig. 2). There also exist two weak C-H···π interactions (Table 1).

### Experimental

For the preparation of the title compound, (I), 2,4-bis(4-methoxyphenyl)-1,3,2,4-dithiadiphosphetane-2,4-disulfide (0.51 g, 1.23 mmol) and (S)-(-)-1-(2-naphthyl)ethanol (0.43 g, 2.46 mmol) were suspended in toluene (20 ml). The mixture was refluxed until all solids had dissolved. The yellow solution was cooled to room temperature, filtered and treated with excess triethyl amine. The product was precipitated at 291 K from hexane/toluene (1:4) as colorless crystals. They were isolated by filtration, washed with n-pentane and dried in air (yield; 0.85 g, 72.64%, m.p. 359-360 K).

### Refinement

H1 atom is located in a difference Fourier synthesis and refined isotropically. The C-bound H-atoms were positioned geometrically with C—H = 0.93, 0.98, 0.97 and 0.96 Å, for aromatic, methine, methylene and methyl H-atoms, respectively, and constrained to ride on their parent atoms, with *U*_iso_(H) = k × *U*_eq_(C), where k = 1.5 for methyl H-atoms and k = 1.2 for all other H-atoms.

### Crystal data

**Table:**

C_6_H_16_N^+^·C_19_H_18_O_2_PS_2_^−^	*F*(000) = 1016
*M**_r_* = 475.62	*D*_x_ = 1.242 Mg m^−^^3^
Orthorhombic, *P*2_1_2_1_2_1_	Mo *K*α radiation, λ = 0.71073 Å
Hall symbol: P 2ac 2ab	Cell parameters from 9895 reflections
*a* = 9.3782 (3) Å	θ = 2.7–28.4°
*b* = 12.3467 (5) Å	µ = 0.29 mm^−^^1^
*c* = 21.9651 (8) Å	*T* = 294 K
*V* = 2543.33 (16) Å^3^	Block, colorless
*Z* = 4	0.52 × 0.36 × 0.32 mm

### Data collection

**Table:**

Bruker Kappa APEXII CCD area-detector diffractometer	6343 independent reflections
Radiation source: fine-focus sealed tube	5946 reflections with *I* > 2σ(*I*)
graphite	*R*_int_ = 0.030
φ and ω scans	θ_max_ = 28.4°, θ_min_ = 1.9°
Absorption correction: multi-scan (*SADABS*; Bruker, 2005)	*h* = −11→12
*T*_min_ = 0.862, *T*_max_ = 0.912	*k* = −15→16
43596 measured reflections	*l* = −29→29

### Refinement

**Table:**

Refinement on *F*^2^	Secondary atom site location: difference Fourier map
Least-squares matrix: full	Hydrogen site location: inferred from neighbouring sites
*R*[*F*^2^ > 2σ(*F*^2^)] = 0.036	H atoms treated by a mixture of independent and constrained refinement
*wR*(*F*^2^) = 0.094	*w* = 1/[σ^2^(*F*_o_^2^) + (0.0477*P*)^2^ + 1.2869*P*] where *P* = (*F*_o_^2^ + 2*F*_c_^2^)/3
*S* = 1.06	(Δ/σ)_max_ < 0.001
6343 reflections	Δρ_max_ = 0.78 e Å^−^^3^
289 parameters	Δρ_min_ = −0.26 e Å^−^^3^
0 restraints	Absolute structure: Flack (1983), 2752 Friedel pairs
Primary atom site location: structure-invariant direct methods	Flack parameter: −0.01 (6)

### Special details

**Table:**

Geometry. All esds (except the esd in the dihedral angle between two l.s. planes) are estimated using the full covariance matrix. The cell esds are taken into account individually in the estimation of esds in distances, angles and torsion angles; correlations between esds in cell parameters are only used when they are defined by crystal symmetry. An approximate (isotropic) treatment of cell esds is used for estimating esds involving l.s. planes.
Refinement. Refinement of F^2^ against ALL reflections. The weighted R-factor wR and goodness of fit S are based on F^2^, conventional R-factors R are based on F, with F set to zero for negative F^2^. The threshold expression of F^2^ > 2sigma(F^2^) is used only for calculating R-factors(gt) etc. and is not relevant to the choice of reflections for refinement. R-factors based on F^2^ are statistically about twice as large as those based on F, and R- factors based on ALL data will be even larger.

### Fractional atomic coordinates and isotropic or equivalent isotropic displacement parameters (Å^2^)

**Table:**

	*x*	*y*	*z*	*U*_iso_*/*U*_eq_	
S1	0.60412 (5)	0.18821 (4)	0.04905 (2)	0.02652 (11)	
S2	0.57588 (5)	0.14708 (4)	0.19936 (2)	0.02634 (11)	
P1	0.48082 (5)	0.18524 (4)	0.12180 (2)	0.01859 (10)	
O1	0.34594 (14)	0.10520 (11)	0.11076 (6)	0.0211 (3)	
O2	0.14963 (18)	0.59555 (13)	0.14733 (7)	0.0327 (3)	
N1	−0.09897 (17)	0.20257 (14)	0.15241 (7)	0.0233 (3)	
H1	−0.187 (3)	0.190 (2)	0.1517 (11)	0.029 (6)*	
C1	0.38210 (18)	0.31092 (15)	0.12904 (8)	0.0192 (3)	
C2	0.3729 (2)	0.36634 (17)	0.18402 (8)	0.0235 (4)	
H2	0.4193	0.3392	0.2182	0.028*	
C3	0.2957 (2)	0.46103 (17)	0.18858 (9)	0.0264 (4)	
H3	0.2904	0.4972	0.2257	0.032*	
C4	0.2256 (2)	0.50278 (16)	0.13774 (9)	0.0235 (4)	
C5	0.2361 (2)	0.44975 (16)	0.08213 (9)	0.0229 (4)	
H5	0.1911	0.4777	0.0478	0.027*	
C6	0.3143 (2)	0.35486 (17)	0.07823 (8)	0.0222 (4)	
H6	0.3217	0.3197	0.0409	0.027*	
C7	0.0639 (3)	0.6348 (2)	0.09831 (11)	0.0393 (5)	
H7A	0.0105	0.6967	0.1117	0.059*	
H7B	−0.0007	0.5790	0.0854	0.059*	
H7C	0.1241	0.6551	0.0649	0.059*	
C8	0.3722 (2)	−0.00862 (16)	0.09988 (10)	0.0261 (4)	
H8	0.4752	−0.0209	0.0970	0.031*	
C9	0.3140 (3)	−0.07059 (18)	0.15388 (11)	0.0331 (5)	
H9A	0.3298	−0.1467	0.1480	0.050*	
H9B	0.2136	−0.0571	0.1576	0.050*	
H9C	0.3617	−0.0473	0.1903	0.050*	
C10	0.3016 (2)	−0.03846 (18)	0.03867 (10)	0.0291 (4)	
C11	0.2350 (2)	0.04142 (18)	0.00213 (10)	0.0295 (4)	
H11	0.2360	0.1134	0.0146	0.035*	
C12	0.1688 (2)	0.01411 (19)	−0.05147 (11)	0.0322 (4)	
H12	0.1235	0.0672	−0.0744	0.039*	
C13	0.1694 (2)	−0.09410 (19)	−0.07195 (10)	0.0308 (4)	
C14	0.1044 (2)	−0.1237 (2)	−0.12876 (11)	0.0358 (5)	
H14	0.0577	−0.0718	−0.1521	0.043*	
C15	0.1116 (3)	−0.2262 (2)	−0.14773 (11)	0.0381 (5)	
H15	0.0704	−0.2448	−0.1848	0.046*	
C16	0.1806 (3)	−0.3082 (2)	−0.11267 (11)	0.0406 (5)	
H16	0.1840	−0.3790	−0.1270	0.049*	
C17	0.2415 (3)	−0.28267 (19)	−0.05816 (11)	0.0363 (5)	
H17	0.2852	−0.3362	−0.0350	0.044*	
C18	0.2382 (2)	−0.17606 (17)	−0.03732 (9)	0.0264 (4)	
C19	0.3031 (2)	−0.14337 (19)	0.01921 (10)	0.0301 (4)	
H19	0.3475	−0.1955	0.0432	0.036*	
C20	−0.0239 (2)	0.14297 (19)	0.10174 (10)	0.0304 (4)	
H20A	0.0780	0.1436	0.1093	0.037*	
H20B	−0.0410	0.1803	0.0636	0.037*	
C21	−0.0742 (3)	0.0270 (2)	0.09624 (13)	0.0425 (6)	
H21A	−0.0452	−0.0130	0.1316	0.064*	
H21B	−0.0330	−0.0053	0.0606	0.064*	
H21C	−0.1763	0.0256	0.0930	0.064*	
C22	−0.0597 (2)	0.16311 (18)	0.21476 (9)	0.0292 (4)	
H22B	−0.0739	0.0854	0.2164	0.035*	
H22A	−0.1235	0.1960	0.2442	0.035*	
C23	0.0930 (2)	0.1883 (2)	0.23275 (10)	0.0356 (5)	
H23A	0.1128	0.1572	0.2719	0.053*	
H23B	0.1060	0.2653	0.2346	0.053*	
H23C	0.1569	0.1582	0.2031	0.053*	
C24	−0.0782 (2)	0.32156 (17)	0.14353 (10)	0.0305 (4)	
H24A	−0.1139	0.3416	0.1036	0.037*	
H24B	0.0230	0.3375	0.1445	0.037*	
C25	−0.1526 (3)	0.3895 (2)	0.19115 (12)	0.0438 (6)	
H25A	−0.1499	0.4643	0.1792	0.066*	
H25B	−0.1051	0.3810	0.2296	0.066*	
H25C	−0.2500	0.3665	0.1949	0.066*	

### Atomic displacement parameters (Å^2^)

**Table:**

	*U*^11^	*U*^22^	*U*^33^	*U*^12^	*U*^13^	*U*^23^
S1	0.0237 (2)	0.0337 (2)	0.0222 (2)	0.0015 (2)	0.00873 (17)	0.0038 (2)
S2	0.0195 (2)	0.0391 (3)	0.0204 (2)	0.00257 (18)	−0.00307 (17)	0.0067 (2)
P1	0.01450 (18)	0.0253 (2)	0.01599 (19)	−0.00017 (17)	0.00075 (15)	0.00270 (18)
O1	0.0184 (6)	0.0224 (6)	0.0225 (6)	−0.0001 (5)	0.0014 (5)	−0.0009 (5)
O2	0.0394 (8)	0.0292 (8)	0.0294 (8)	0.0085 (6)	−0.0055 (6)	−0.0048 (6)
N1	0.0169 (7)	0.0286 (9)	0.0245 (8)	−0.0022 (6)	0.0012 (6)	0.0043 (6)
C1	0.0180 (7)	0.0228 (8)	0.0167 (8)	−0.0016 (7)	−0.0001 (6)	0.0010 (7)
C2	0.0263 (9)	0.0282 (10)	0.0161 (8)	−0.0030 (7)	−0.0046 (6)	0.0013 (7)
C3	0.0322 (10)	0.0281 (10)	0.0190 (9)	−0.0016 (8)	−0.0033 (7)	−0.0057 (7)
C4	0.0233 (9)	0.0222 (9)	0.0248 (10)	−0.0021 (7)	−0.0008 (7)	−0.0013 (7)
C5	0.0236 (9)	0.0276 (10)	0.0176 (9)	0.0016 (7)	−0.0029 (7)	0.0024 (7)
C6	0.0227 (8)	0.0288 (9)	0.0150 (8)	0.0007 (7)	0.0002 (6)	−0.0009 (7)
C7	0.0469 (14)	0.0349 (12)	0.0360 (12)	0.0139 (11)	−0.0034 (10)	0.0013 (10)
C8	0.0228 (9)	0.0237 (9)	0.0317 (10)	0.0011 (7)	0.0058 (8)	−0.0022 (8)
C9	0.0378 (12)	0.0277 (11)	0.0338 (11)	0.0008 (9)	0.0010 (9)	0.0060 (9)
C10	0.0249 (9)	0.0300 (10)	0.0325 (11)	−0.0059 (8)	0.0099 (8)	−0.0050 (9)
C11	0.0312 (10)	0.0282 (10)	0.0292 (11)	0.0023 (8)	0.0028 (8)	−0.0004 (8)
C12	0.0323 (10)	0.0318 (11)	0.0324 (11)	0.0051 (9)	0.0000 (9)	0.0051 (9)
C13	0.0269 (10)	0.0309 (11)	0.0346 (11)	0.0006 (8)	0.0049 (8)	0.0011 (9)
C14	0.0275 (10)	0.0462 (13)	0.0336 (11)	−0.0018 (9)	−0.0005 (9)	−0.0022 (10)
C15	0.0341 (11)	0.0497 (14)	0.0304 (11)	−0.0024 (10)	−0.0062 (9)	−0.0061 (10)
C16	0.0425 (13)	0.0359 (12)	0.0432 (13)	−0.0048 (11)	−0.0009 (10)	−0.0022 (11)
C17	0.0392 (12)	0.0289 (11)	0.0409 (13)	0.0010 (9)	−0.0019 (10)	−0.0011 (9)
C18	0.0200 (8)	0.0276 (10)	0.0316 (10)	−0.0024 (7)	0.0021 (7)	0.0047 (8)
C19	0.0261 (10)	0.0301 (10)	0.0341 (11)	0.0026 (8)	−0.0016 (8)	0.0056 (9)
C20	0.0208 (9)	0.0390 (11)	0.0315 (10)	−0.0016 (8)	0.0026 (7)	−0.0054 (9)
C21	0.0301 (11)	0.0376 (13)	0.0597 (16)	0.0023 (10)	−0.0012 (11)	−0.0117 (11)
C22	0.0237 (9)	0.0366 (12)	0.0271 (9)	−0.0038 (8)	−0.0005 (7)	0.0099 (8)
C23	0.0259 (10)	0.0507 (13)	0.0302 (10)	−0.0059 (10)	−0.0052 (8)	0.0083 (10)
C24	0.0340 (10)	0.0279 (10)	0.0295 (9)	−0.0025 (9)	0.0022 (8)	0.0040 (8)
C25	0.0553 (15)	0.0315 (12)	0.0445 (14)	0.0017 (11)	0.0031 (12)	−0.0052 (11)

### Geometric parameters (Å, °)

**Table:**

S1—P1	1.9726 (6)	C11—H11	0.9300
S2—P1	1.9798 (6)	C12—C13	1.410 (3)
P1—O1	1.6234 (14)	C12—H12	0.9300
P1—C1	1.8140 (19)	C13—C14	1.436 (3)
O1—C8	1.447 (2)	C13—C18	1.421 (3)
O2—C4	1.365 (2)	C14—C15	1.335 (4)
O2—C7	1.429 (3)	C14—H14	0.9300
N1—C20	1.509 (3)	C15—C16	1.427 (4)
N1—C22	1.500 (2)	C15—H15	0.9300
N1—C24	1.495 (3)	C16—C17	1.364 (3)
N1—H1	0.84 (3)	C16—H16	0.9300
C1—C2	1.391 (3)	C17—C18	1.394 (3)
C2—C3	1.379 (3)	C17—H17	0.9300
C2—H2	0.9300	C18—C19	1.441 (3)
C3—H3	0.9300	C19—H19	0.9300
C4—C3	1.395 (3)	C20—H20A	0.9700
C4—C5	1.390 (3)	C20—H20B	0.9700
C5—C6	1.385 (3)	C21—C20	1.513 (3)
C5—H5	0.9300	C21—H21A	0.9600
C6—C1	1.395 (2)	C21—H21B	0.9600
C6—H6	0.9300	C21—H21C	0.9600
C7—H7A	0.9600	C22—C23	1.518 (3)
C7—H7B	0.9600	C22—H22A	0.9700
C7—H7C	0.9600	C22—H22B	0.9700
C8—C9	1.513 (3)	C23—H23A	0.9600
C8—C10	1.543 (3)	C23—H23B	0.9600
C8—H8	0.9800	C23—H23C	0.9600
C9—H9A	0.9600	C24—H24A	0.9700
C9—H9B	0.9600	C24—H24B	0.9700
C9—H9C	0.9600	C25—C24	1.512 (3)
C10—C11	1.417 (3)	C25—H25A	0.9600
C10—C19	1.364 (3)	C25—H25B	0.9600
C11—C12	1.373 (3)	C25—H25C	0.9600
			
S1—P1—S2	115.95 (3)	C13—C12—H12	119.8
O1—P1—S1	110.31 (5)	C12—C13—C14	121.1 (2)
O1—P1—S2	109.54 (5)	C12—C13—C18	120.4 (2)
O1—P1—C1	97.83 (8)	C18—C13—C14	118.5 (2)
C1—P1—S1	110.75 (6)	C13—C14—H14	120.3
C1—P1—S2	110.97 (6)	C15—C14—C13	119.4 (2)
C8—O1—P1	118.90 (12)	C15—C14—H14	120.3
C4—O2—C7	117.52 (17)	C14—C15—C16	121.8 (2)
C20—N1—H1	110.5 (18)	C14—C15—H15	119.1
C22—N1—C20	113.63 (17)	C16—C15—H15	119.1
C22—N1—H1	101.4 (17)	C15—C16—H16	120.0
C24—N1—C20	108.81 (16)	C17—C16—C15	120.0 (2)
C24—N1—C22	113.99 (16)	C17—C16—H16	120.0
C24—N1—H1	108.2 (18)	C16—C17—C18	119.8 (2)
C2—C1—P1	121.93 (14)	C16—C17—H17	120.1
C2—C1—C6	118.37 (17)	C18—C17—H17	120.1
C6—C1—P1	119.70 (14)	C13—C18—C19	117.0 (2)
C1—C2—H2	119.6	C17—C18—C13	120.5 (2)
C3—C2—C1	120.87 (17)	C17—C18—C19	122.5 (2)
C3—C2—H2	119.6	C10—C19—C18	122.1 (2)
C2—C3—C4	120.19 (18)	C10—C19—H19	118.9
C2—C3—H3	119.9	C18—C19—H19	118.9
C4—C3—H3	119.9	N1—C20—C21	112.04 (19)
O2—C4—C3	115.63 (17)	N1—C20—H20A	109.2
O2—C4—C5	124.61 (18)	N1—C20—H20B	109.2
C5—C4—C3	119.75 (18)	C21—C20—H20A	109.2
C4—C5—H5	120.3	C21—C20—H20B	109.2
C6—C5—C4	119.39 (17)	H20A—C20—H20B	107.9
C6—C5—H5	120.3	C20—C21—H21B	109.5
C1—C6—H6	119.3	C20—C21—H21C	109.5
C5—C6—C1	121.41 (17)	C20—C21—H21A	109.5
C5—C6—H6	119.3	H21B—C21—H21A	109.5
O2—C7—H7A	109.5	H21B—C21—H21C	109.5
O2—C7—H7B	109.5	H21C—C21—H21A	109.5
O2—C7—H7C	109.5	N1—C22—C23	113.76 (17)
H7A—C7—H7B	109.5	N1—C22—H22B	108.8
H7A—C7—H7C	109.5	N1—C22—H22A	108.8
H7B—C7—H7C	109.5	C23—C22—H22A	108.8
O1—C8—C9	107.47 (16)	C23—C22—H22B	108.8
O1—C8—C10	107.62 (16)	H22B—C22—H22A	107.7
O1—C8—H8	109.2	C22—C23—H23A	109.5
C9—C8—C10	114.04 (18)	C22—C23—H23B	109.5
C9—C8—H8	109.2	C22—C23—H23C	109.5
C10—C8—H8	109.2	H23A—C23—H23B	109.5
C8—C9—H9A	109.5	H23A—C23—H23C	109.5
C8—C9—H9B	109.5	H23B—C23—H23C	109.5
C8—C9—H9C	109.5	N1—C24—C25	113.29 (18)
H9A—C9—H9B	109.5	N1—C24—H24A	108.9
H9A—C9—H9C	109.5	N1—C24—H24B	108.9
H9B—C9—H9C	109.5	C25—C24—H24A	108.9
C11—C10—C8	121.10 (18)	C25—C24—H24B	108.9
C19—C10—C8	119.7 (2)	H24A—C24—H24B	107.7
C19—C10—C11	119.2 (2)	C24—C25—H25A	109.5
C10—C11—H11	119.5	C24—C25—H25B	109.5
C12—C11—C10	120.9 (2)	C24—C25—H25C	109.5
C12—C11—H11	119.5	H25A—C25—H25B	109.5
C11—C12—C13	120.3 (2)	H25A—C25—H25C	109.5
C11—C12—H12	119.8	H25B—C25—H25C	109.5
			
S1—P1—O1—C8	62.17 (14)	C5—C6—C1—P1	179.00 (15)
S2—P1—O1—C8	−66.62 (14)	C5—C6—C1—C2	−1.7 (3)
C1—P1—O1—C8	177.78 (14)	O1—C8—C10—C11	3.9 (2)
S1—P1—C1—C2	−133.20 (14)	O1—C8—C10—C19	−176.08 (17)
S1—P1—C1—C6	46.06 (16)	C9—C8—C10—C11	123.0 (2)
S2—P1—C1—C2	−2.94 (17)	C9—C8—C10—C19	−57.0 (3)
S2—P1—C1—C6	176.32 (13)	C8—C10—C11—C12	−178.18 (19)
O1—P1—C1—C2	111.53 (16)	C19—C10—C11—C12	1.8 (3)
O1—P1—C1—C6	−69.20 (16)	C8—C10—C19—C18	179.61 (18)
P1—O1—C8—C9	113.03 (16)	C11—C10—C19—C18	−0.4 (3)
P1—O1—C8—C10	−123.74 (14)	C10—C11—C12—C13	−1.7 (3)
C7—O2—C4—C3	−173.5 (2)	C11—C12—C13—C14	−178.2 (2)
C7—O2—C4—C5	6.5 (3)	C11—C12—C13—C18	0.1 (3)
C22—N1—C20—C21	68.7 (2)	C12—C13—C14—C15	177.6 (2)
C24—N1—C20—C21	−163.15 (19)	C18—C13—C14—C15	−0.7 (3)
C20—N1—C22—C23	68.3 (2)	C12—C13—C18—C17	−178.6 (2)
C24—N1—C22—C23	−57.1 (2)	C12—C13—C18—C19	1.2 (3)
C20—N1—C24—C25	178.55 (19)	C14—C13—C18—C17	−0.3 (3)
C22—N1—C24—C25	−53.5 (2)	C14—C13—C18—C19	179.57 (19)
P1—C1—C2—C3	−179.18 (16)	C13—C14—C15—C16	0.9 (4)
C6—C1—C2—C3	1.6 (3)	C14—C15—C16—C17	0.0 (4)
C1—C2—C3—C4	−0.1 (3)	C15—C16—C17—C18	−1.0 (4)
O2—C4—C3—C2	178.63 (18)	C16—C17—C18—C13	1.2 (3)
C5—C4—C3—C2	−1.3 (3)	C16—C17—C18—C19	−178.7 (2)
O2—C4—C5—C6	−178.79 (19)	C13—C18—C19—C10	−1.1 (3)
C3—C4—C5—C6	1.1 (3)	C17—C18—C19—C10	178.7 (2)
C4—C5—C6—C1	0.4 (3)		

### Hydrogen-bond geometry (Å, °)

**Table:**

Cg1 and Cg2 are the centroids of the C1–C6 and C10–C13/C18/C19 rings, respectively.

**Table:**

*D*—H···*A*	*D*—H	H···*A*	*D*···*A*	*D*—H···*A*
N1—H1···S2^i^	0.84 (3)	2.52 (3)	3.2911 (17)	154 (2)
C20—H20A···O1	0.97	2.56	3.505 (2)	166
C7—H7B···Cg2^ii^	0.96	2.90	3.6578 (28)	137
C24—H24B···Cg1^iii^	0.97	2.79	3.7496 (24)	171

Symmetry codes: (i) *x*−1, *y*, *z*; (ii) −*x*−1, *y*+1/2, −*z*+1/2; (iii) *x*+1, *y*, *z*.
